# Supplementary figures and images for: A Single-Cell Transcriptional Roadmap of the Mouse and Human Lymph Node Lymphatic Vasculature
Source: Front Cardiovasc Med. 2020 Apr 30;7:52. doi: 10.3389/fcvm.2020.00052 (PMC7204639; doi:10.3389/fcvm.2020.00052)

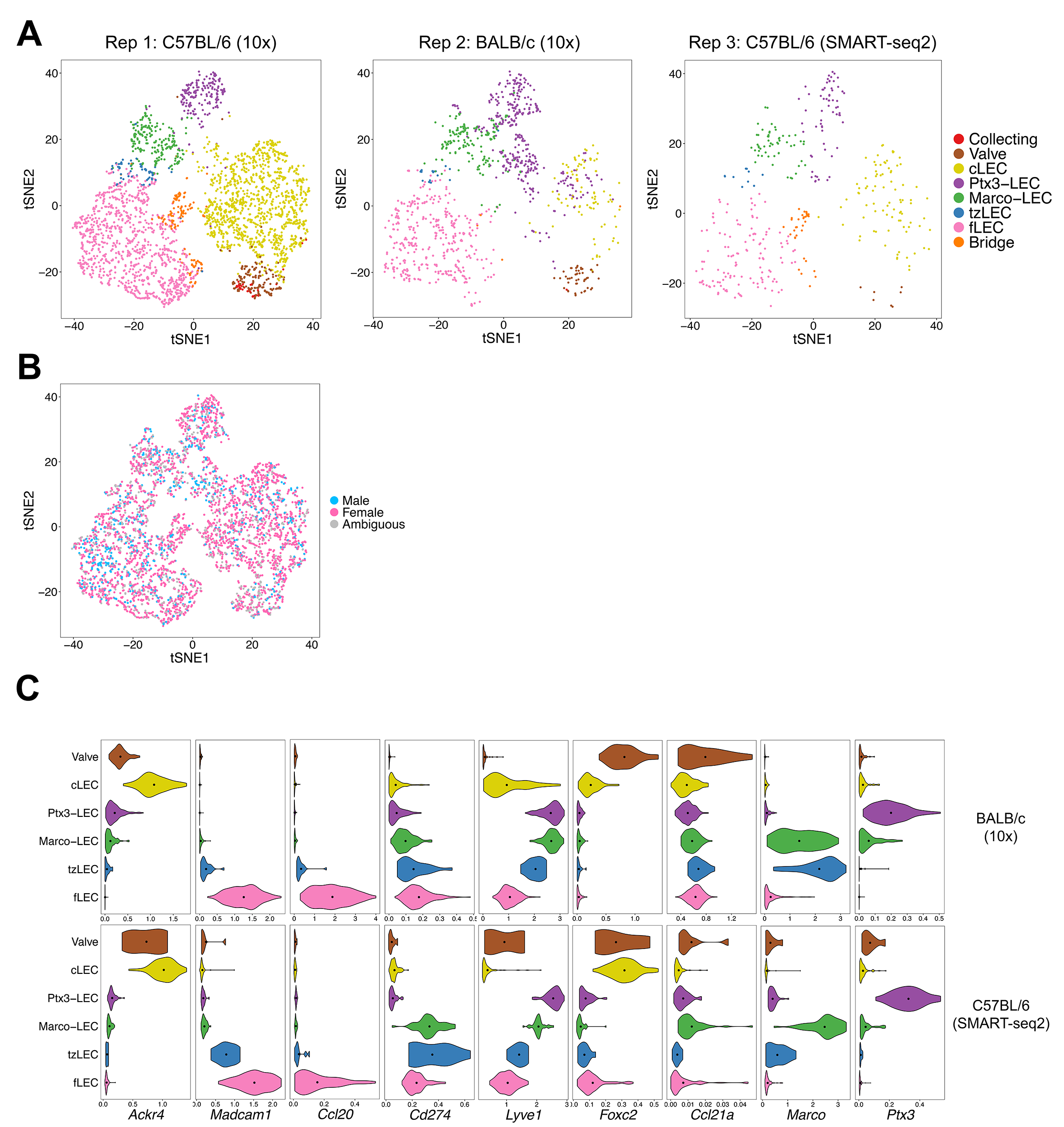

Supplement: Figure S1 — Mouse LN LEC subsets in biological replicates. (A) tSNE plots of LEC from three individual biological replicates, colored by subset. (B) tSNE plot of LEC from all replicates, colored by sex. (C) Expression of subset defining genes in BALB/c (10x) and C57BL/6 (SMART-seq2) mice. Dots indicate mean log-normalized transcript count. [file Image_1.TIF]

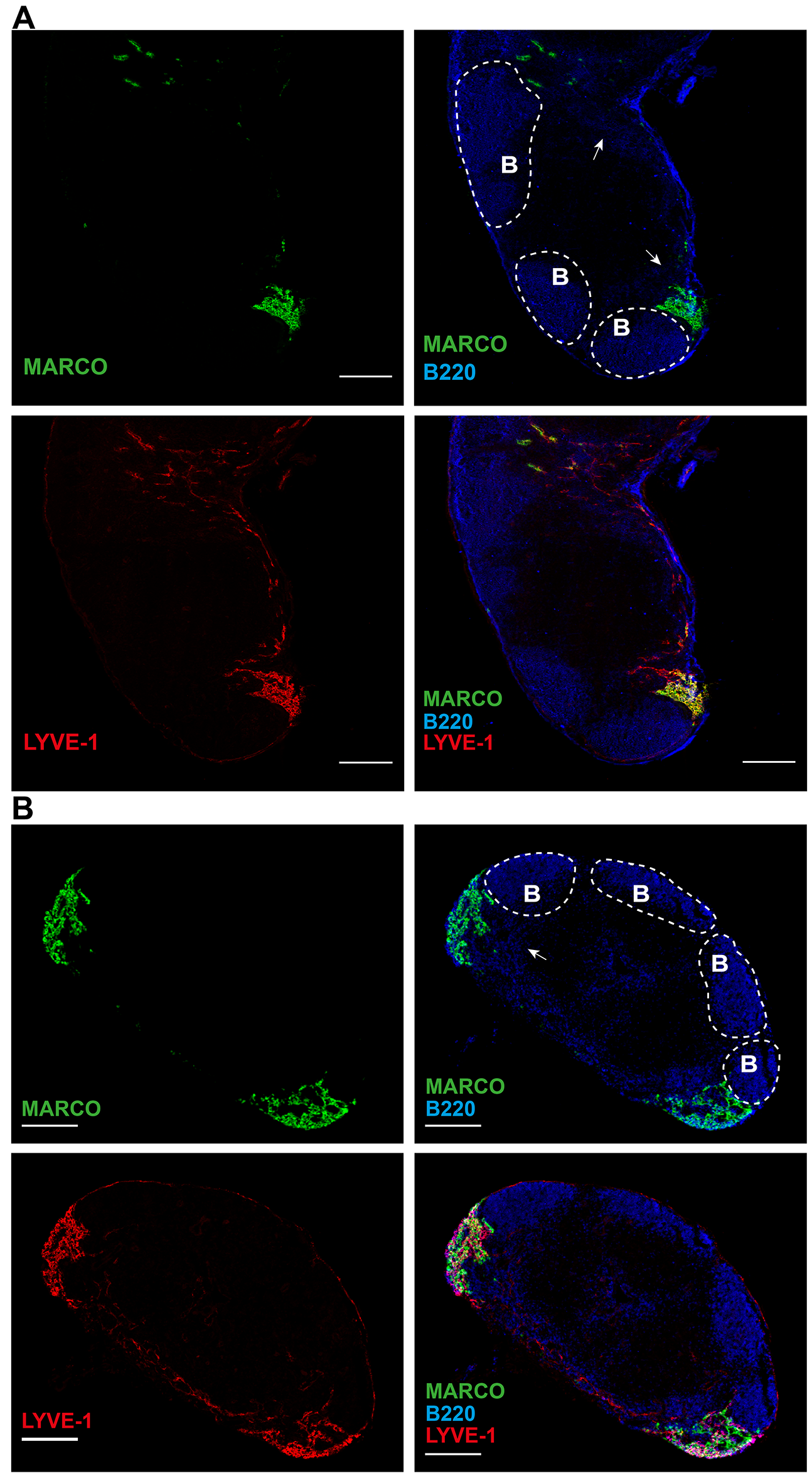

Supplement: Figure S2 — MARCO+ LECs are close to B-cell follicular area. B220 (blue), LYVE-1 (red), and MARCO (green) in inguinal (A) and popliteal (B) LN sections of wild type mice. B-cell follicular areas (white dashed lines) and scattered B-lineage plasma cells in the medulla (white arrows) are indicated. Composite images are made by overlay of serial sections in ImageJ. Data are representative of three or more independent experiments. Scale bar = 200 μm. [file Image_2.TIF]

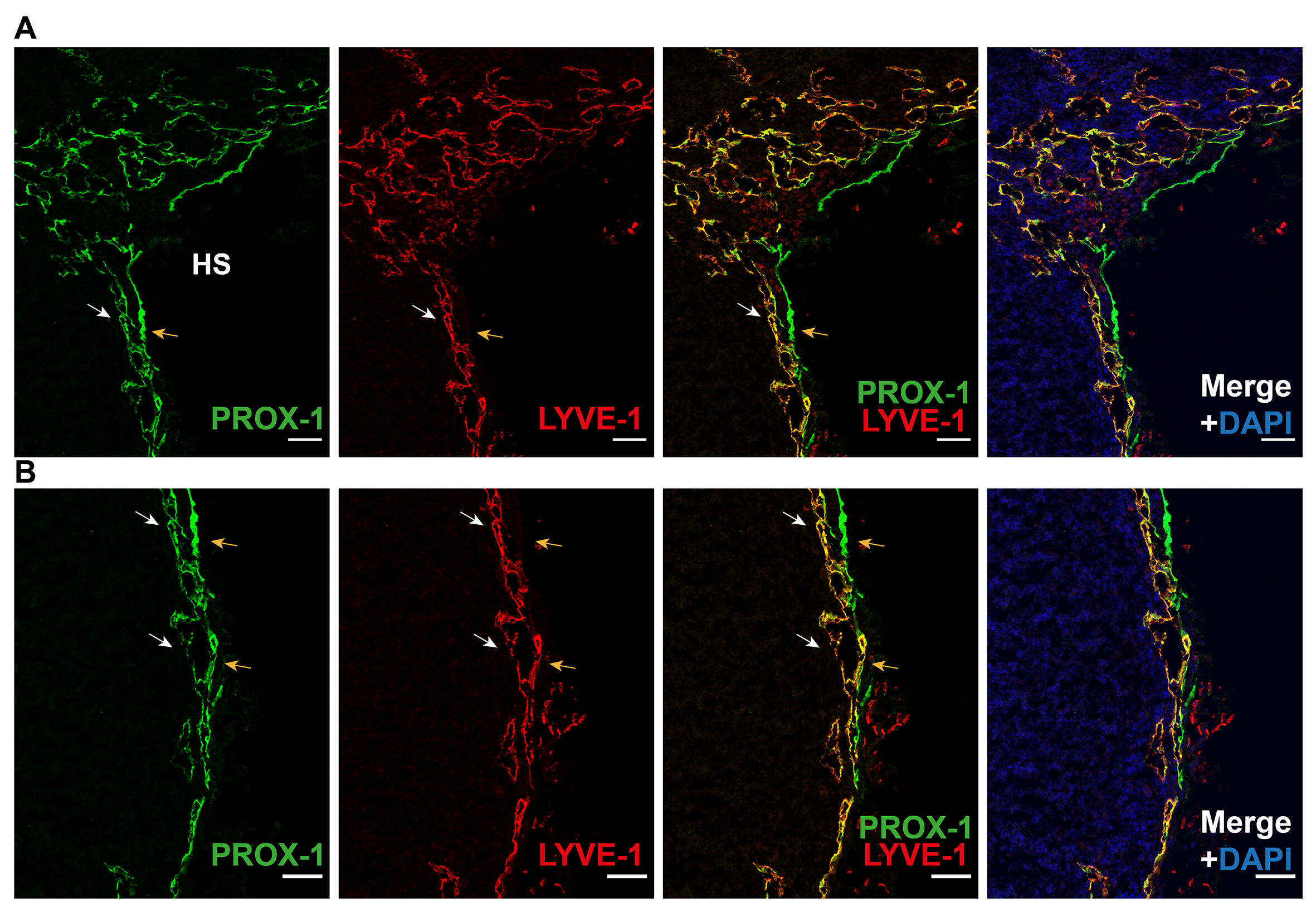

Supplement: Figure S3 — Interaction between Ptx3-LECs and cLECs in peri-hilar sinuses. (A) Hilus and (B) Perihilar region. Inguinal LNs from Prox1-GFP transgenic mouse stained for GFP (PROX-1) (green) and LYVE-1 (red), counterstained with DAPI (blue). HS = hilus. LYVE-1+ LECs (Ptx3-LEC area) are indicated with white arrows and LYVE-1− cLECs are indicated with orange arrows. Data are representative of three or more independent experiments. Scale bar = 50 μm. [file Image_3.TIF]

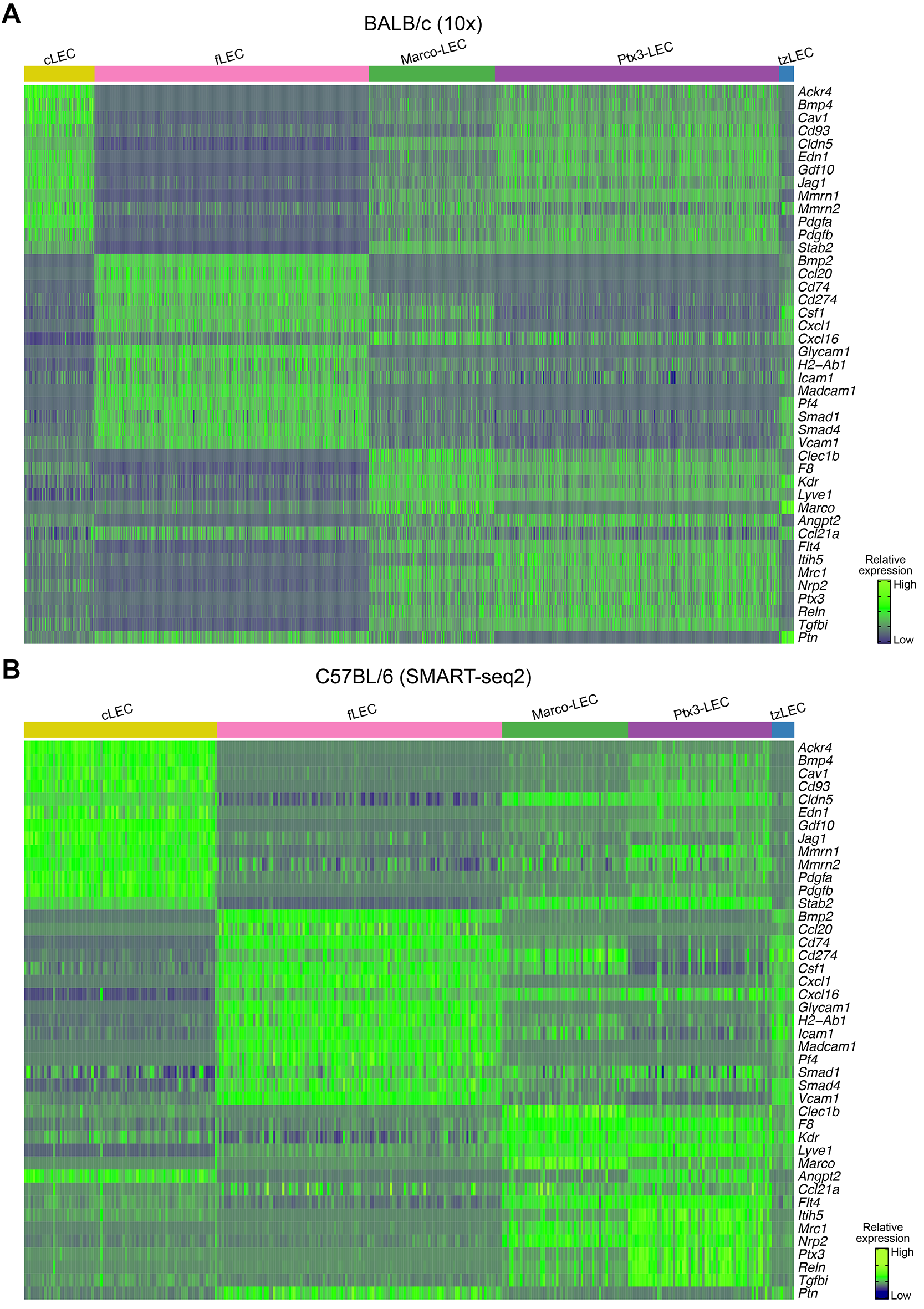

Supplement: Figure S4 — Heatmaps of LEC subset DEGs in biological replicates. Heatmaps of select DEGs in LECs of (A) BALB/c (10x) and (B) C57BL/6 (SMART-seq2) mice. Values are imputed log counts (row scaled). [file Image_4.TIF]

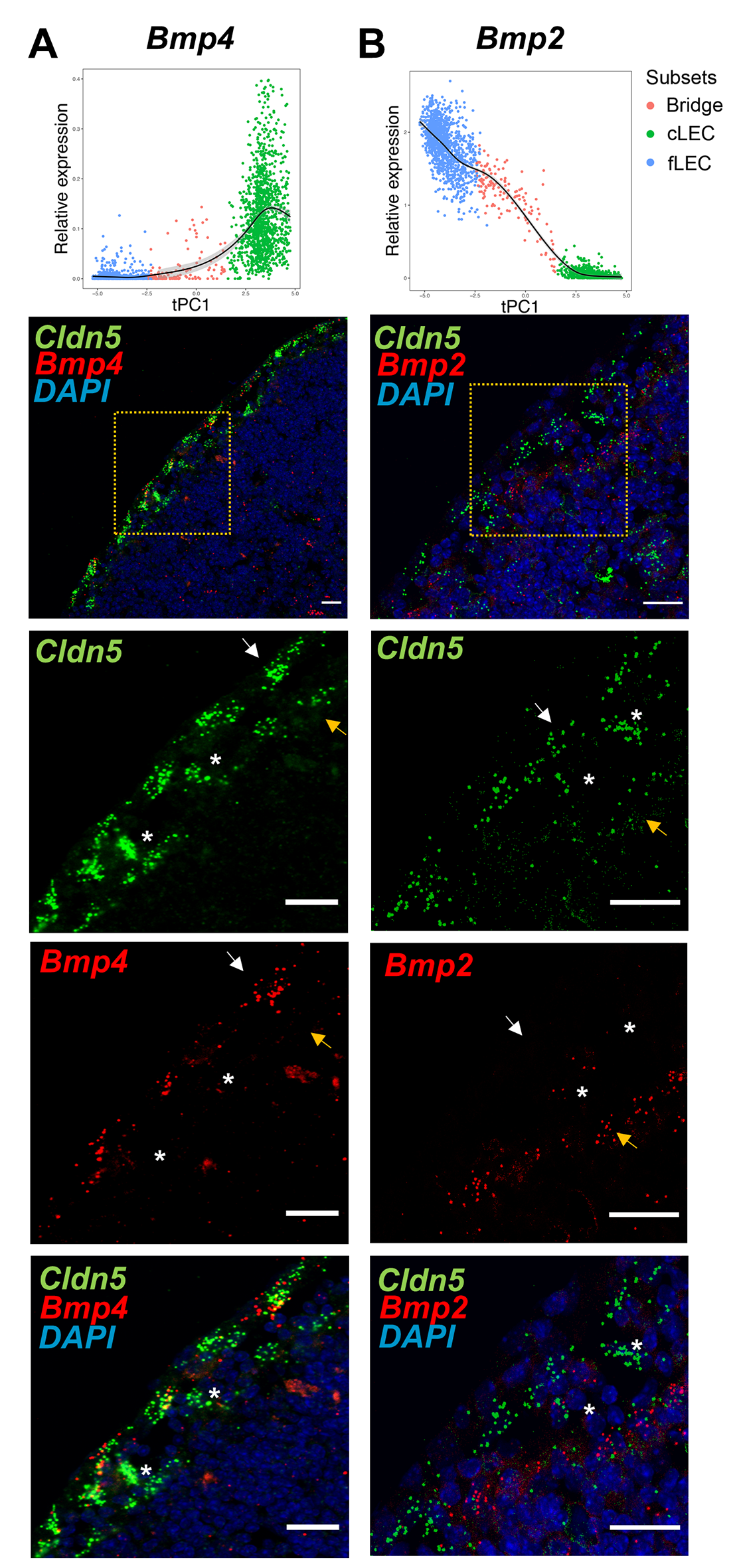

Supplement: Figure S5 — Bmp4 and Bmp2 expression by cLECs, fLECs and SCS bridging cells. In situ hybridization (RNAscope-ISH) of mouse inguinal LNs. (A) mRNA detection of Cldn5 (green) and Bmp4 (red) with fluorescent probes. ROI inset (orange dotted box) shown below. (B) mRNA detection of Cldn5 (green) and Bmp2 (red) with fluorescent probes. ROI inset (orage dotted box) shown below. Counterstain is DAPI (blue). The ceiling lymphatic endothelial cells (cLECs) (white arrows) and the lymphatics endothelium lining the floor (fLECs) (orange arrows) are indicated. Bridges are indicated with white stars. scRNA-seq expression across cLEC, bridge and fLEC populations is shown above. Outliers are not shown. Scale bar = 20 μm. [file Image_5.TIF]

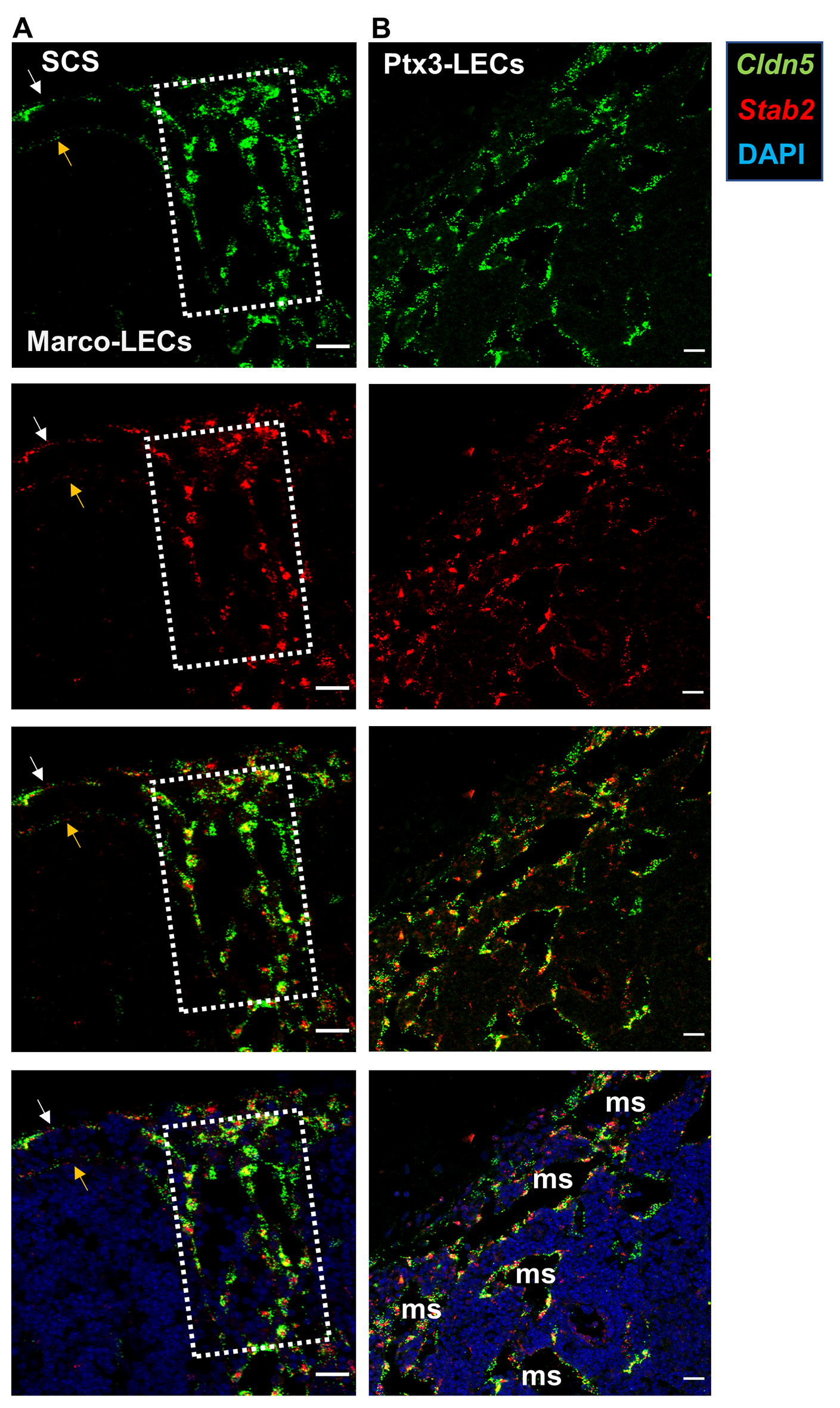

Supplement: Figure S6 — Low Stabilin 2 expression differentiates fLECs from medullary populations and cLECs. In situ hybridization (RNAscope-ISH) of mouse inguinal LNs. Detection of Cldn5 (green) and Stab2 (red) mRNA with fluorescent probes, counterstained with DAPI (blue). (A) Subcapsular sinus area; cLECs (white arrows) and fLECs (orange arrows) populations are indicated. Peri-follicular medulla (corresponding to Marco-LECs) is outlined with white dotted rectangle. (B) Central medulla (peri-hilar) on the efferent (eff) side of the LN (Ptx3-LECs). The medullary sinuses (ms) are indicated. Scale bar = 20 μm. [file Image_6.TIF]

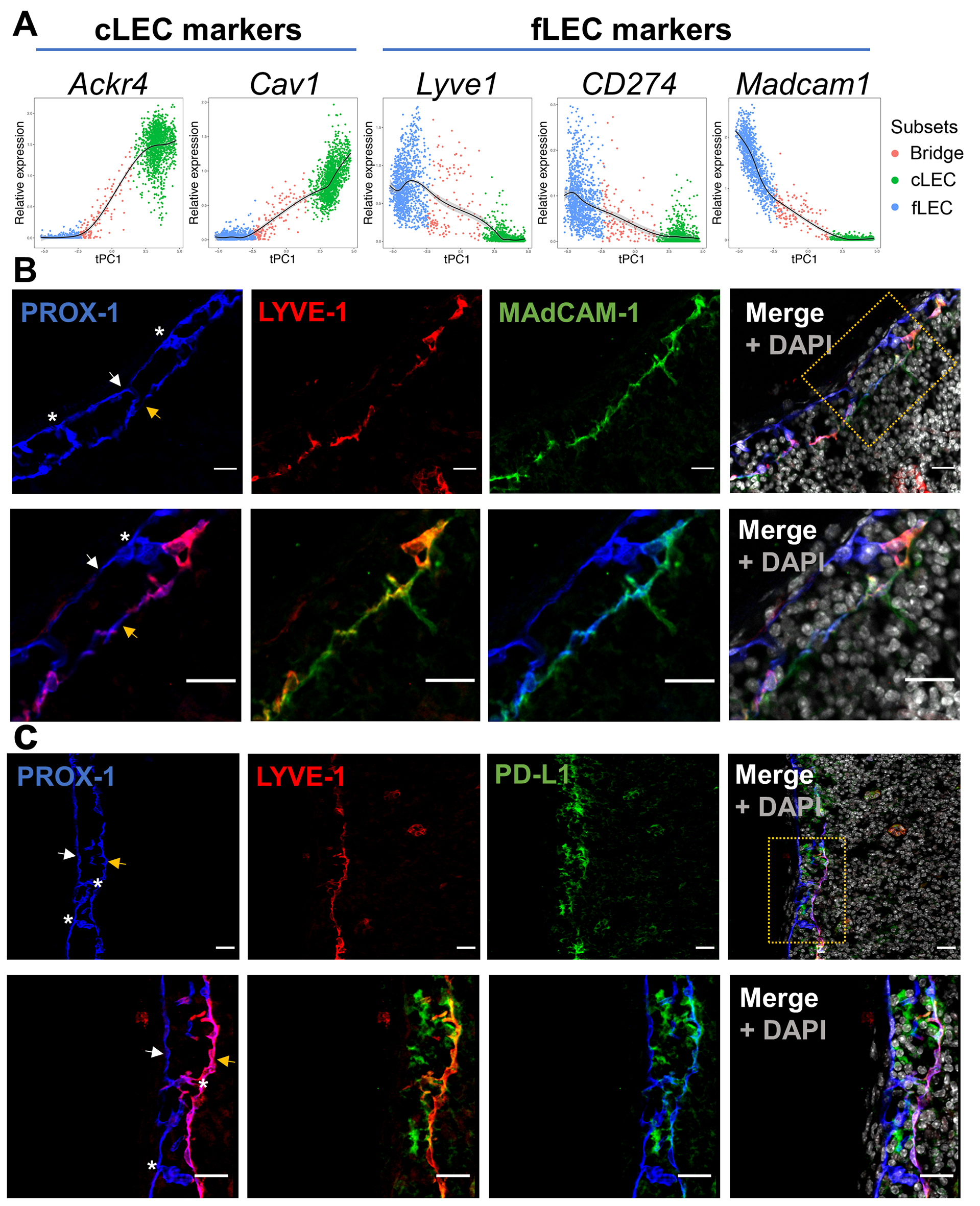

Supplement: Figure S7 — SCS bridging cells. (A) scRNA-seq expression of cLEC and fLEC marker genes across cLEC, bridge, and fLEC populations. Outliers are not shown. (B,C) Immunoreactivity of GFP (PROX-1-GFP) (blue), LYVE-1(red) and MAdCAM-1 (B) or PD-L1 (C) (green), in inguinal LNs from Prox1-GFP transgenic mice, counterstained with DAPI (gray). Area of insets is shown by orange dotted rectangle. The ceiling lymphatic endothelial cells (cLECs) (white arrows), the lymphatic endothelium lining the floor (fLECs) (orange arrows) and bridge population (white stars) are indicated. Data are representative of three or more independent experiment. Scale bar = 20 μm. [file Image_7.TIF]

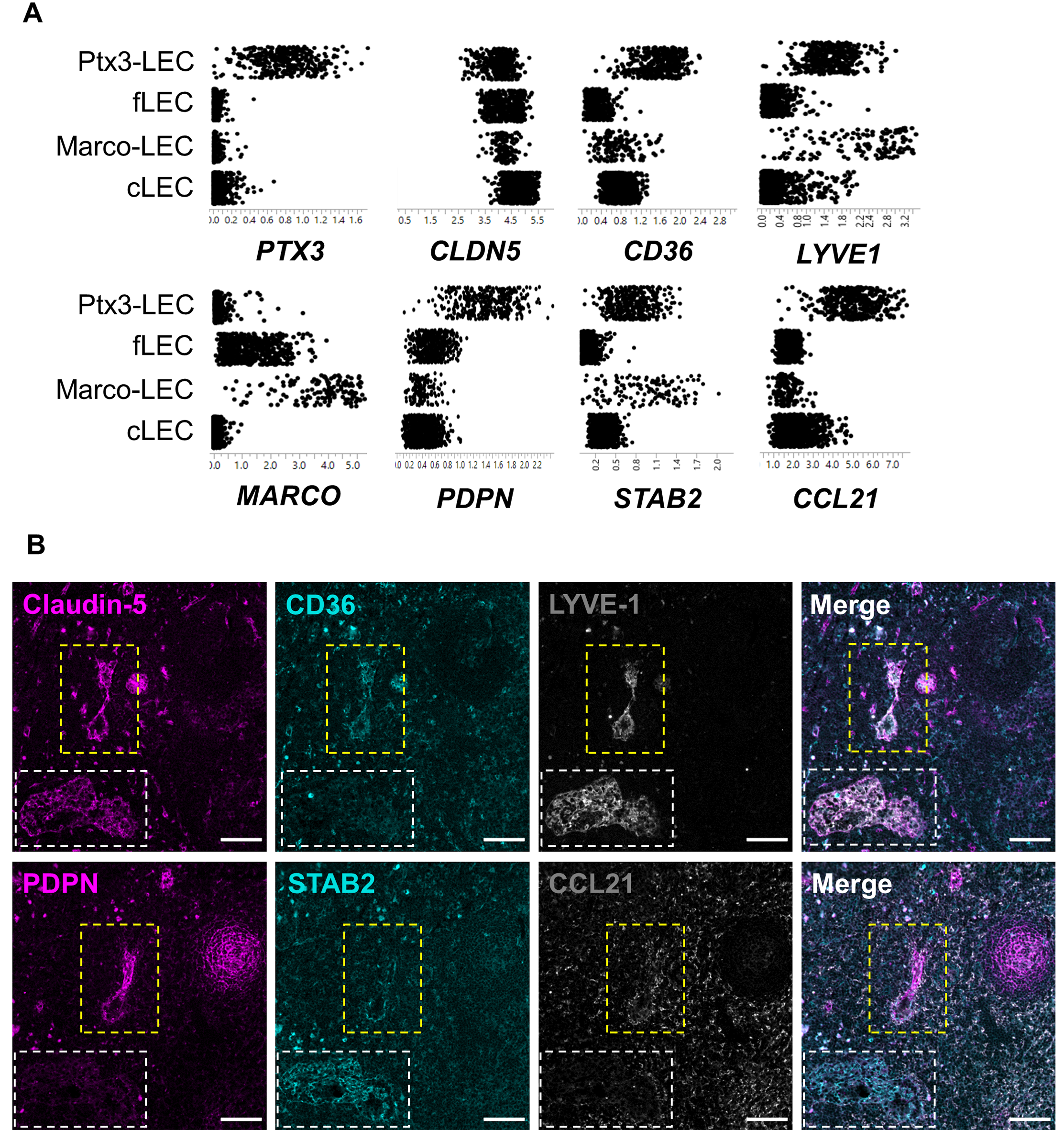

Supplement: Figure S8 — Human PTX3 and MARCO subsets. (A) Gene expression of selected genes in human Ptx3-LECs, fLECs, Marco-LECs, and cLECs subsets. Dots indicate log-normalized transcript counts. (B) Immunofluorescence staining of Ptx3-LECs and Marco-LECs in a formalin-fixed, paraffin-embedded human axillary LN. Yellow dashed lined box: PTX3 subset marked by high Claudin-5, CD36, PDPN, intermediate LYVE-1, STAB2 and CCL21. White dashed lined box: MARCO subset marked by high Claudin-5, LYVE-1, STAB2, low PDPN, and CCL21. Scale bar: 100 μm. [file Image_8.TIF]

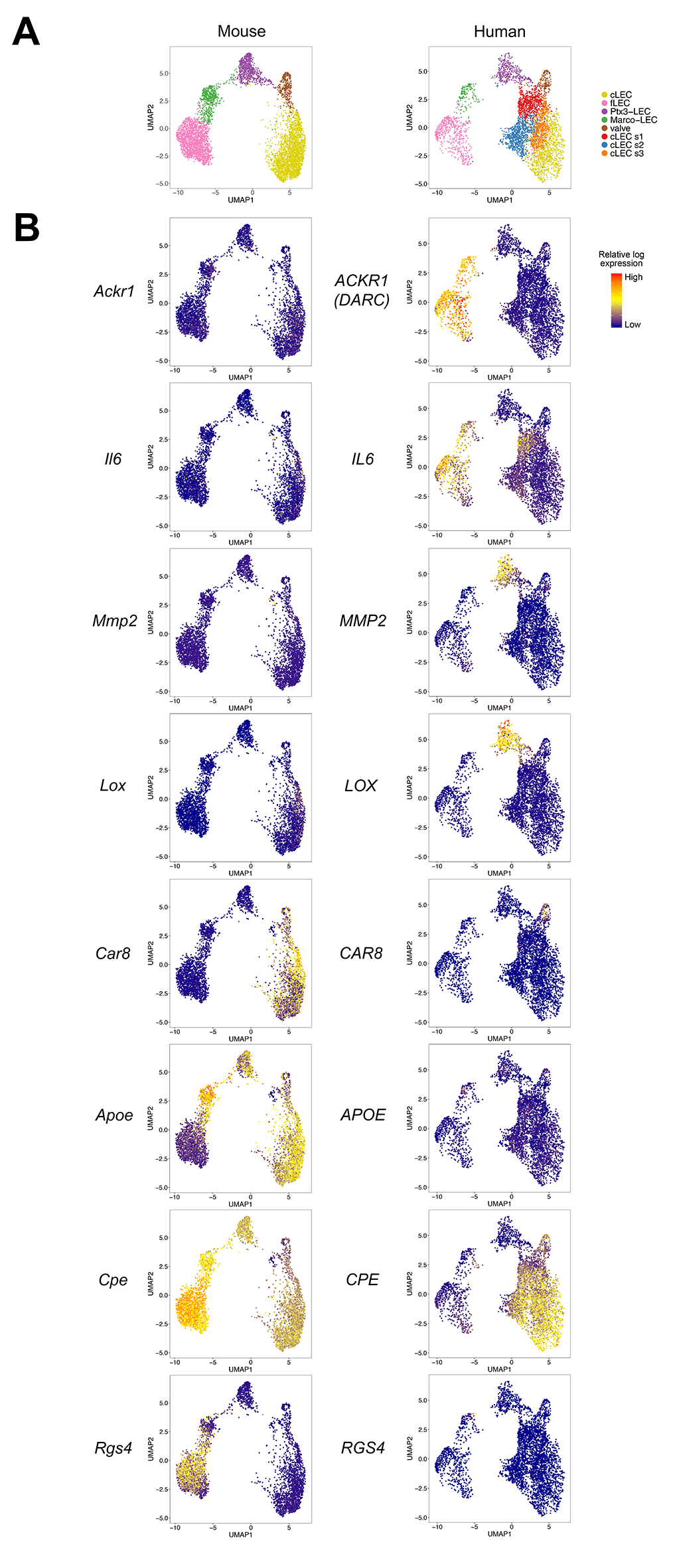

Supplement: Figure S9 — Illustration of differential gene expression patterns in mouse and human. (A) UMAP of aligned mouse and human LEC, colored by subset (reproduced from Figure 7A). (B) Expression pattern of indicated genes, projected on UMAP plot of mouse (left) and human (right) LN LECs. Values are imputed log counts. [file Image_9.TIF]
